# Postoperative Corticosteroid Use After Same-Day Versus Staged Septoplasty With Functional Endoscopic Sinus Surgery: Does Surgical Timing Matter?

**DOI:** 10.7759/cureus.114969

**Published:** 2026-08-22

**Authors:** Khalid Mohamed, Jonathan Vuillier, Nabil Agag, Ronak Bhatia, Fatima Ahmed, Ahmad Mohammed, Elizabeth Beyene, Mekdem Bisrat, Austin Yap, Miriam Micheal

**Affiliations:** 1 Otolaryngology - Head and Neck Surgery, Howard University College of Medicine, Washington, USA; 2 Medical School, Kansas City University College of Osteopathic Medicine, Kansas City, USA; 3 Medical School, Howard University College of Medicine, Washington DC, USA; 4 Internal Medicine, St. George’s University School of Medicine, St. George’s, GRD; 5 Internal Medicine, Howard University Hospital, Washington DC, USA; 6 Otolaryngology - Head and Neck Surgery, MedStar Georgetown University Hospital, Washington DC, USA; 7 Internal Medicine, Howard University College of Medicine, Washingon DC, USA; 8 Internal Medicine, University of Maryland School of Medicine, Baltimore, USA

**Keywords:** corticosteroids, functional endoscopic sinus surgery, nasal polyps, septoplasty, surgical timing

## Abstract

Background

Septoplasty is frequently performed with functional endoscopic sinus surgery (FESS), either during the same operative session or as a staged procedure. Whether same-day versus staged septoplasty with FESS is associated with differences in postoperative corticosteroid use or postoperative nasal polyp diagnosis remains unclear.

Objective

The study aimed to compare postoperative corticosteroid use and postoperative nasal polyp diagnosis after same-day versus staged septoplasty with FESS.

Methods

We performed a retrospective cohort study using the TriNetX U.S. Collaborative Network. Adults undergoing both septoplasty and FESS were categorized into same-day and staged cohorts, with staged procedures separated by at least 30 days. One-to-one propensity score matching balanced age, sex, race, baseline nasal polyp diagnosis, and baseline systemic and intranasal corticosteroid use. The index date was the FESS procedure date. Postoperative corticosteroid use was assessed from 15 to 180 days after FESS, and postoperative nasal polyp diagnosis was assessed from 15 to 730 days.

Results

After matching, 3,171 patients remained in each cohort. Systemic corticosteroid use occurred in 925 same-day patients (29.17%) and 825 staged patients (26.01%; risk difference, 3.15%; 95% confidence interval (CI), 0.96%-5.35%; odds ratio (OR), 1.17; 95% CI, 1.05-1.31; p = 0.0050). Intranasal corticosteroid use occurred in 1,283 same-day patients (40.46%) and 1,070 staged patients (33.74%; risk difference, 6.72%; 95% CI, 4.35%-9.09%; OR, 1.33; 95% CI, 1.21-1.48; p < 0.0001). Postoperative nasal polyp diagnosis did not differ significantly between cohorts (28.82% vs 26.96%; hazard ratio, 1.08; 95% CI, 0.98-1.18; log-rank p = 0.1189).

Conclusions

Compared with staged surgery, same-day surgery was associated with significantly higher postoperative systemic and intranasal corticosteroid prescribing, but there was no statistically significant difference in postoperative nasal polyp diagnosis between cohorts. These findings are hypothesis-generating and should not be interpreted as evidence that staging reduces total corticosteroid burden or provides equivalent endoscopic disease control. Further prospective studies with standardized medication capture, symptom scores, and endoscopic follow-up are needed to clarify whether surgical timing influences postoperative inflammatory control after septoplasty and FESS.

## Introduction

Nasal polyps are benign inflammatory growths of the nasal passages and paranasal sinuses [1]. They are commonly associated with chronic rhinosinusitis, a condition characterized by persistent sinonasal mucosal inflammation, nasal obstruction, facial pressure, hyposmia or anosmia, and impaired quality of life [2,3]. Although medical therapy can reduce polyp burden and symptoms, chronic rhinosinusitis with nasal polyps often requires long-term anti-inflammatory management, including after surgery [4].

Treatment typically begins with intranasal corticosteroids, which are used to reduce mucosal inflammation and polyp size [5]. Short courses of systemic corticosteroids may also be used for symptom flares or perioperative disease control [6]. However, repeated or cumulative systemic corticosteroid exposure is associated with clinically important adverse effects, including hyperglycemia, hypertension, weight gain, mood disturbance, adrenal suppression, osteoporosis, and infection risk [7]. Therefore, strategies that reduce avoidable corticosteroid exposure while maintaining sinonasal disease control are clinically relevant.

Functional endoscopic sinus surgery (FESS) is commonly performed when symptoms or inflammatory burden persist despite medical therapy. Septoplasty may be performed concurrently to correct septal deviation, improve surgical access, and optimize postoperative nasal airflow [8-12]. In practice, septoplasty and FESS may be performed during the same operative session or staged on separate dates. This decision may reflect anatomy, disease severity, surgeon preference, patient preference, insurance or reimbursement considerations, and logistical factors. Whether surgical timing is associated with differences in postoperative medication use remains uncertain.

Postoperative nasal polyp diagnosis is difficult to interpret in large electronic health record datasets because diagnosis codes may represent new disease, persistent disease, recurrence, or carried-forward coding rather than endoscopically confirmed recurrent polyposis. For this reason, postoperative corticosteroid prescribing may reflect postoperative management intensity, while coded nasal polyp diagnosis should be interpreted cautiously. In this retrospective cohort study, we evaluated whether same-day versus staged septoplasty and FESS was associated with postoperative corticosteroid prescribing and coded postoperative nasal polyp diagnosis in a large multicenter real-world dataset. The primary outcome was recorded postoperative corticosteroid exposure, including systemic and intranasal corticosteroid prescriptions, and the secondary outcome was coded postoperative nasal polyp diagnosis.

## Materials and methods

Study design and data source

We conducted a retrospective observational cohort study using the TriNetX U.S. Collaborative Network, a federated electronic health record research network. The cohort diagram generated from the TriNetX query identified 67 participating U.S. healthcare organizations and a source network of 114,340,210 patients. TriNetX contains de-identified structured data, including demographics, diagnoses, procedures, medications, and encounters. Because this study used de-identified data and did not involve direct patient contact, institutional review board approval was not required. No manual imputation was performed. Analyses were limited to available structured diagnosis, procedure, and medication-order data in TriNetX; absence of a code was interpreted as absence of recorded evidence in the dataset rather than confirmed clinical absence.

Cohort definition

The study population included adults aged 18 years or older who underwent both septoplasty and FESS. Septoplasty was identified using Current Procedural Terminology (CPT) code 30520. FESS was identified using CPT codes 31254, 31255, 31256, 31267, 31276, 31287, and 31288, corresponding to endoscopic ethmoidectomy, maxillary antrostomy with or without tissue removal, frontal sinus exploration, sphenoidotomy, and sphenoidotomy with tissue removal. The same-day cohort included patients who underwent septoplasty and FESS on the same calendar date. The staged cohort included patients who underwent septoplasty followed by FESS at least 30 days later. The index date for both cohorts was the FESS procedure date. Inclusion criteria are presented in Table 1. No upper interval limit was imposed because no established cutoff defines the maximum interval for staged septoplasty and FESS.

**Table 1:** Inclusion Criteria Used to Define the Study Cohorts CPT: Current Procedural Terminology

Inclusion criterion	Code(s)
Age ≥ 18 years old	N/A
Septoplasty	CPT: 30520
Functional Endoscopic Sinus Surgery (FESS)	CPT: 31254
	CPT: 31255
	CPT: 31256
	CPT: 31267
	CPT: 31276
	CPT: 31287
	CPT: 31288

Patients with conditions that could independently affect nasal polyp diagnosis or postoperative management were excluded. Exclusion criteria included cystic fibrosis, congenital airway or craniofacial abnormalities, systemic vasculitic or inflammatory disorders involving the sinuses, aspirin or analgesic hypersensitivity, and malignant neoplasms of the nasal cavity or paranasal sinuses. Exclusion criteria are presented in Table 2.

**Table 2:** Exclusion Criteria Applied to the Study Cohorts ICD: International Classification of Diseases; NOS: Not Otherwise Specified

Coding system	Code	Diagnosis description
ICD-10-CM	E84	Cystic fibrosis
ICD-10-CM	Q33.4	Congenital bronchiectasis
ICD-10-CM	Q89.8	Other specified congenital malformations
ICD-10-CM	M30.1	Polyarteritis with lung involvement
ICD-10-CM	Z88.6	Allergy status to analgesic agent
ICD-10-CM	C30.0	Malignant neoplasm of nasal cavity
ICD-10-CM	C31.9	Malignant neoplasm of accessory sinus, unspecified
ICD-10-CM	C31.1	Malignant neoplasm of ethmoidal sinus
ICD-10-CM	C31.8	Malignant neoplasm of overlapping sites of accessory sinuses
ICD-10-CM	C31.3	Malignant neoplasm of sphenoid sinus
ICD-10-CM	C31.0	Malignant neoplasm of maxillary sinus
ICD-10-CM	C31.2	Malignant neoplasm of frontal sinus
ICD-O	C31.0	Maxillary sinus
ICD-O	C31.1	Ethmoid sinus
ICD-O	C31.9	Accessory sinus, NOS
ICD-O	C31.3	Sphenoid sinus

Follow-up windows

Follow-up began 15 days after FESS to reduce capture of perioperative medication administration, discharge prescriptions, or documentation related to the surgical encounter. Postoperative corticosteroid use was assessed from 15 to 180 days after FESS to capture early outpatient prescribing beyond the immediate postoperative period. Coded postoperative nasal polyp diagnosis was assessed from 15 to 730 days after FESS to capture longer-term structured diagnosis coding after completion of sinus surgery.

Propensity score matching

One-to-one propensity score matching was performed within TriNetX using its built-in nearest-neighbor algorithm with a caliper of 0.1 pooled standard deviations of the logit of the propensity score. Covariates included age at index, sex, race, baseline nasal polyp diagnosis, baseline systemic corticosteroid use, and baseline intranasal corticosteroid use, all assessed before the index event. Age at index refers to age on the FESS index date rather than current age at the time of analysis. Baseline nasal polyp diagnosis and baseline corticosteroid use were included as available markers of underlying disease severity because they may influence both surgical timing and postoperative corticosteroid prescribing. Covariate balance was evaluated using standardized mean differences, with values less than 0.10 considered adequately balanced.

Outcomes

The primary outcomes were postoperative systemic corticosteroid use and postoperative intranasal corticosteroid use from 15 to 180 days after FESS. Systemic corticosteroids included prednisone (RxNorm 8640), methylprednisolone (RxNorm 6902), and dexamethasone (RxNorm 3264). Intranasal corticosteroids included fluticasone (RxNorm 41126), mometasone (RxNorm 108118), budesonide (RxNorm 19831), beclomethasone (RxNorm 1347), and triamcinolone (RxNorm 10759). Medication exposure was identified from electronic health record prescription data and should be interpreted as prescribing or ordering rather than confirmed medication adherence.

The secondary outcome was coded postoperative nasal polyp diagnosis from 15 to 730 days after FESS, defined using International Classification of Diseases (ICD) ICD-10-CM codes J33.0, J33.1, J33.8, and J33.9. This endpoint should be interpreted as a structured diagnosis-code outcome rather than endoscopically confirmed polyp recurrence because the TriNetX output did not include operative notes, nasal endoscopy findings, imaging scores, or chart-level validation.

Statistical analysis

Risk differences, risk ratios, and odds ratios with 95% confidence intervals were calculated for postoperative corticosteroid use. For coded postoperative nasal polyp diagnosis, between-group differences were assessed using risk estimates as well as Kaplan-Meier analysis with log-rank testing. Cox proportional hazards modeling was used to estimate hazard ratios with 95% confidence intervals. Analyses were conducted within the TriNetX platform. Statistical tests were two-sided, and p < 0.05 was considered statistically significant.

**Figure 1:**
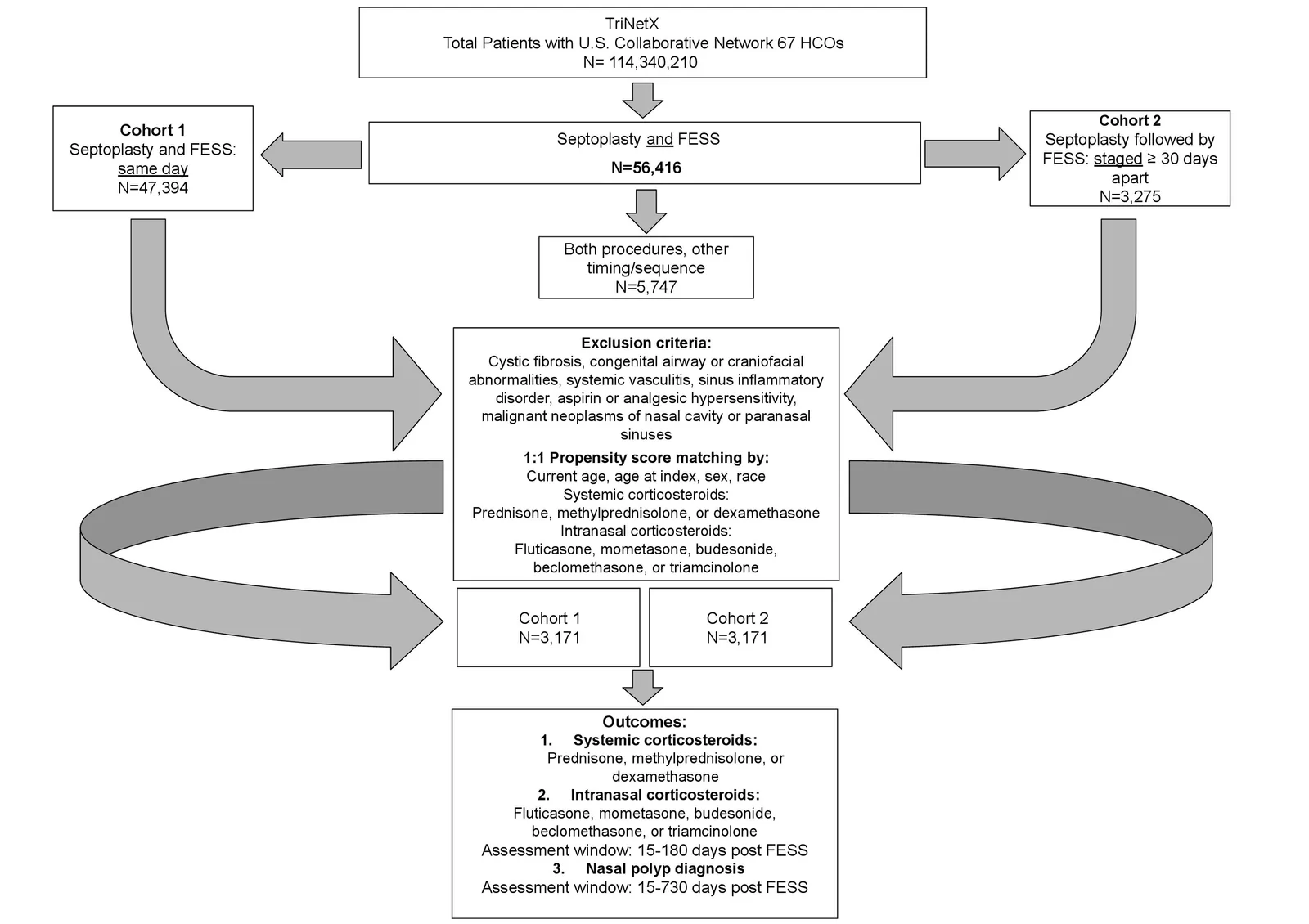
Cohort Selection Flow Diagram Flow diagram showing the patient selection process for same-day and staged septoplasty and FESS cohorts, including propensity score matching and exclusion criteria. The “Both procedures, other timing/sequence” group includes patients with both septoplasty and FESS recorded who did not meet criteria for either comparison cohort. FESS: functional endoscopic sinus surgery; HCO: healthcare organization

The final cohorts were derived by applying the same-day and staged timing definitions, exclusion criteria, and one-to-one propensity score matching. The cohort selection process is shown in Figure 1.

## Results

Cohort selection and matching

The TriNetX US Collaborative Network included 114,340,210 patients across 67 healthcare organizations in the submitted cohort diagram. Among patients undergoing both septoplasty and FESS, 47,394 underwent same-day surgery, and 3,275 underwent staged surgery with septoplasty followed by FESS at least 30 days later. After exclusions and 1:1 propensity score matching, 3,171 patients remained in each cohort. Baseline demographic and clinical characteristics were well balanced after matching, with standardized mean differences below 0.10 for all listed matching variables (Table 3).

**Table 3:** Baseline Characteristics After Propensity Score Matching

Characteristic	Same-day cohort (n = 3,171)	Staged cohort (n = 3,171)	Standardized mean difference
Age, years, mean +/- SD	54.1 +/- 16.3	53.7 +/- 16.4	0.0272
Age at index, years, mean +/- SD	47.8 +/- 16.3	47.4 +/- 16.3	0.0256
Female sex, n (%)	1,529 (48.218%)	1,534 (48.376%)	0.0032
Male sex, n (%)	1,638 (51.656%)	1,637 (51.624%)	0.0006
White race, n (%)	2,355 (74.267%)	2,349 (74.078%)	0.0043
Black or African American race, n (%)	250 (7.884%)	271 (8.546%)	0.0241
Nasal polyp, unspecified, n (%)	990 (31.22%)	1,031 (32.513%)	0.0278
Other polyp of sinus, n (%)	561 (17.692%)	611 (19.268%)	0.0406
Polypoid sinus degeneration, n (%)	37 (1.167%)	49 (1.545%)	0.0327
Polyp of nasal cavity, n (%)	340 (10.722%)	361 (11.384%)	0.0211
Dexamethasone, n (%)	2,052 (64.711%)	1,993 (62.851%)	0.0387
Fluticasone, n (%)	1,923 (60.643%)	1,854 (58.467%)	0.0443
Prednisone, n (%)	1,839 (57.994%)	1,809 (57.048%)	0.0191
Methylprednisolone, n (%)	1,286 (40.555%)	1,289 (40.65%)	0.0019
Budesonide, n (%)	975 (30.747%)	1,043 (32.892%)	0.0461
Triamcinolone, n (%)	794 (25.039%)	802 (25.292%)	0.0058
Mometasone, n (%)	649 (20.467%)	621 (19.584%)	0.0221
Beclomethasone, n (%)	168 (5.298%)	179 (5.645%)	0.0153

Postoperative corticosteroid use

Systemic corticosteroid use within 15-180 days after FESS occurred in 925 of 3,171 same-day patients (29.17%) and 825 of 3,171 staged patients (26.01%). The risk difference was 3.15% (95% CI, 0.96%-5.35%; p = 0.0050), the risk ratio was 1.121 (95% CI, 1.035-1.214), and the odds ratio was 1.171 (95% CI, 1.049-1.308).

**Figure 2:**
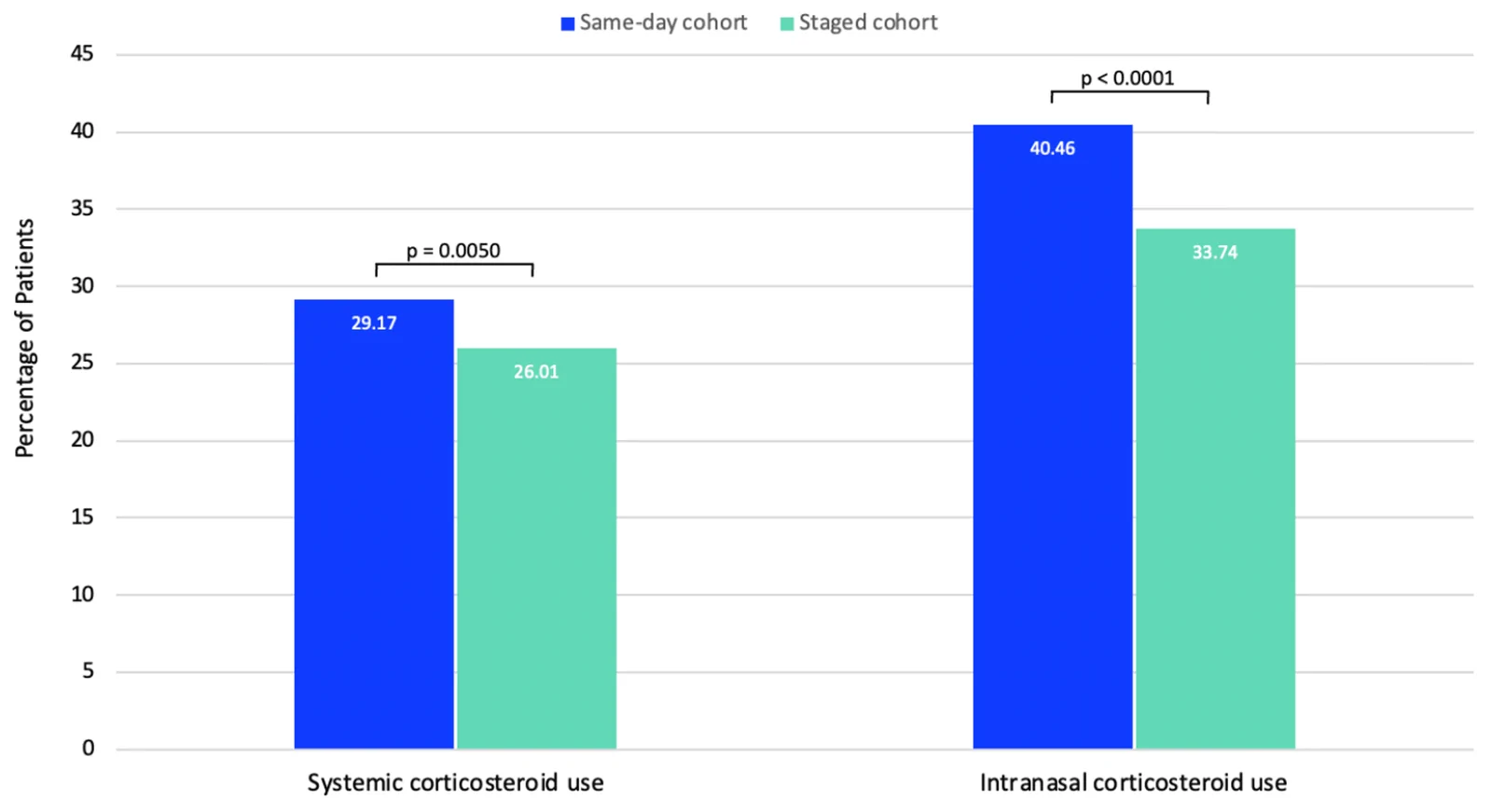
Postoperative Corticosteroid Use After Same-Day Versus Staged Septoplasty and FESS Percentage of propensity score-matched patients with systemic or intranasal corticosteroid use 15-180 days after FESS in the same-day and staged cohorts. P-values indicate between-group comparisons. FESS: functional endoscopic sinus surgery

Intranasal corticosteroid use occurred in 1,283 of 3,171 same-day patients (40.46%) and 1,070 of 3,171 staged patients (33.74%). The risk difference was 6.72% (95% CI, 4.35%-9.09%; p < 0.0001), the risk ratio was 1.199 (95% CI, 1.124-1.279), and the odds ratio was 1.334 (95% CI, 1.205-1.478). These postoperative corticosteroid outcomes are summarized in Table 4 and Figure 2.

**Table 4:** Association Between Surgical Timing and Postoperative Outcomes

Outcome	Same-Day cohort (n=3,171)	Staged cohort (n=3,171)	Risk Difference % (95% CI)	Risk Ratio (95% CI)	Odds Ratio (95% CI)	p-value
Systemic corticosteroid use	925 (29.17%)	825 (26.01%)	3.15 (0.96 to 5.35)	1.121 (1.035–1.214)	1.171 (1.049–1.308)	0.0050
Intranasal corticosteroid use	1,283 (40.46%)	1,070 (33.74%)	6.72 (4.35 to 9.09)	1.199 (1.124–1.279)	1.334 (1.205–1.478)	<0.0001
Post-op nasal polyp diagnosis	914 (28.82%)	855 (26.96%)	1.86 (−0.35 to 4.07)	1.069 (0.988–1.157)	1.097 (0.983–1.224)	0.0985

Coded postoperative nasal polyp diagnosis

**Figure 3:**
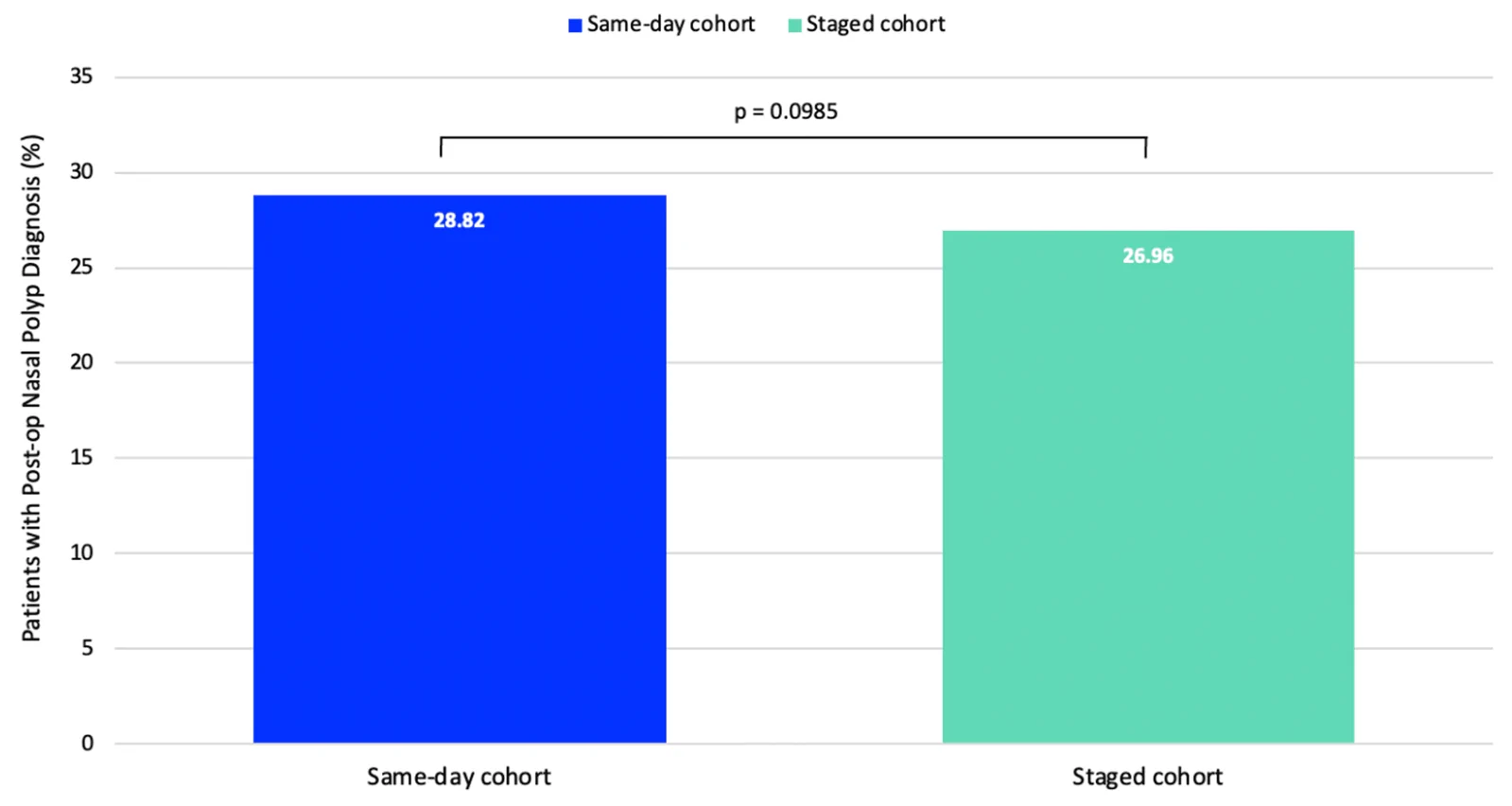
Postoperative Nasal Polyp Diagnosis After Same-Day Versus Staged Septoplasty and FESS Percentage of propensity score-matched patients with coded postoperative nasal polyp diagnosis 15-730 days after FESS in the same-day and staged cohorts. P-value indicates between-group comparisons. FESS: functional endoscopic sinus surgery

Coded postoperative nasal polyp diagnosis within 15-730 days after FESS occurred in 914 of 3,171 same-day patients (28.82%) and 855 of 3,171 staged patients (26.96%). The risk difference was 1.86% (95% CI, -0.35% to 4.07%; p = 0.0985), the risk ratio was 1.069 (95% CI, 0.988-1.157), and the odds ratio was 1.097 (95% CI, 0.983-1.224). These findings are summarized in Table 4 and Figure 3.

**Figure 4:**
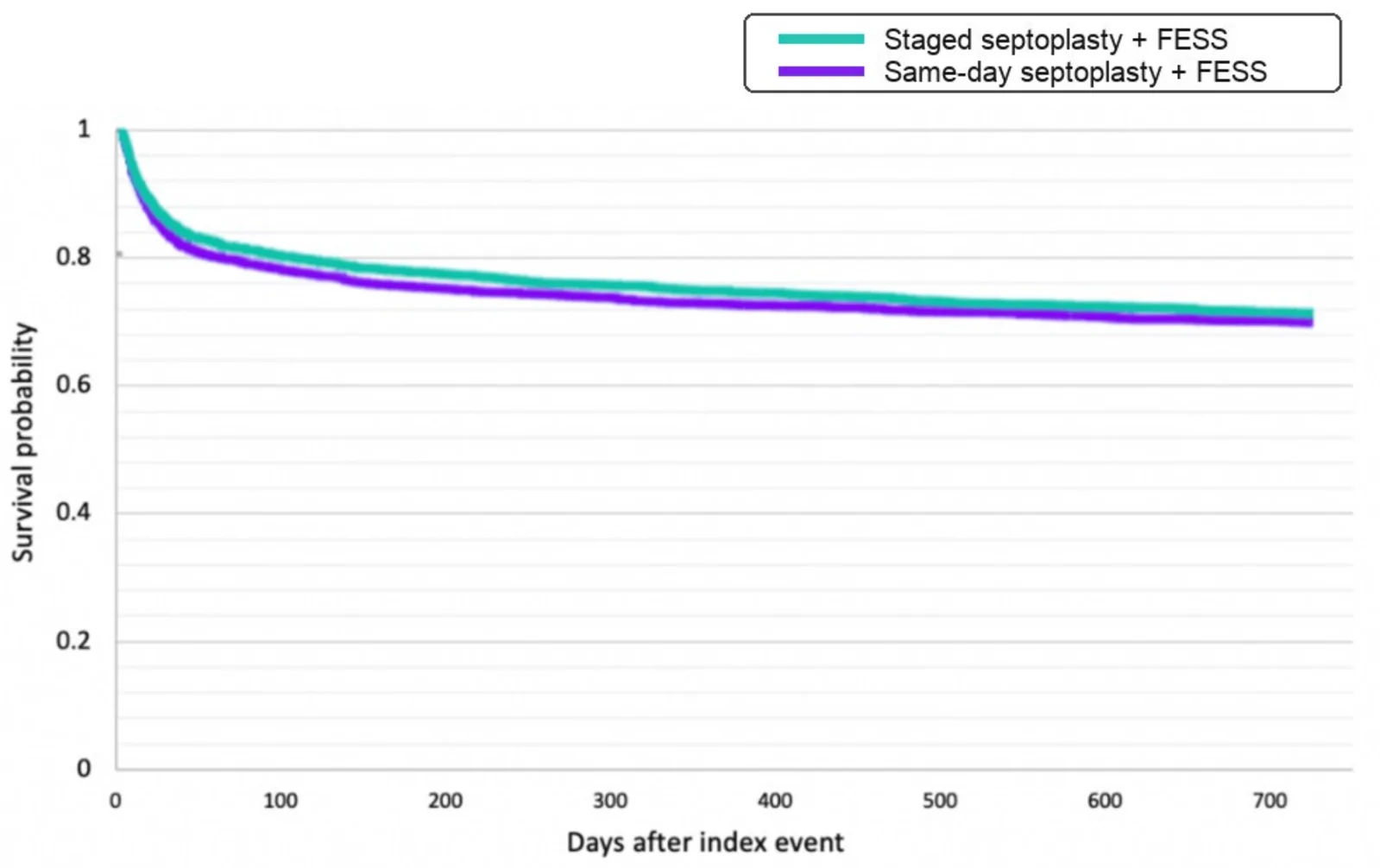
Kaplan-Meier Analysis of Coded Postoperative Nasal Polyp Diagnosis After Same-Day Versus Staged Septoplasty and FESS Kaplan-Meier curve comparing time to coded postoperative nasal polyp diagnosis from 15 to 730 days after FESS in the propensity score-matched same-day and staged cohorts. The hazard ratio was 1.077 (95% CI, 0.981-1.182), with no statistically significant between-group difference by log-rank testing (p = 0.1189).

Kaplan-Meier analysis showed no statistically significant difference between cohorts for time to coded postoperative nasal polyp diagnosis (hazard ratio, 1.077; 95% CI, 0.981-1.182; log-rank p = 0.1189) (Figure 4).

## Discussion

In this large propensity score-matched retrospective cohort study, the same-day performance of septoplasty and FESS was associated with higher observed postoperative corticosteroid prescribing than staged surgery. Both systemic and intranasal corticosteroid prescribing were more frequent in the same-day cohort during the 15-180-day post-FESS window. In contrast, coded postoperative nasal polyp diagnosis within two years did not differ significantly between cohorts, and Kaplan-Meier analysis similarly showed no significant difference in time to coded postoperative nasal polyp diagnosis.

These findings should be interpreted cautiously. The observed difference in corticosteroid prescribing does not prove that same-day surgery causes greater postoperative inflammation or that staged surgery reduces total steroid burden. Surgical timing may reflect unmeasured clinical factors, including disease severity, anatomy, surgeon preference, patient preference, insurance or reimbursement considerations, and local postoperative management patterns. Same-day surgery may also be chosen for patients in whom concurrent correction of septal deviation improves surgical access or nasal airflow. Therefore, the association should be viewed as a signal of different postoperative medication patterns rather than a causal comparison of surgical strategies.

The absence of a significant difference in coded postoperative nasal polyp diagnosis also requires caution. In electronic health record data, a postoperative nasal polyp code may represent new disease, persistent disease, recurrence, or carried-forward diagnosis coding. The database does not provide endoscopic findings, polyp grade, Lund-Kennedy scores, Lund-Mackay computed tomography scores, symptom scores, operative findings, or chart-level confirmation. As a result, this endpoint should not be interpreted as definitive evidence of equivalent endoscopic disease control.

The clinical relevance of postoperative corticosteroid prescribing depends on whether prescribing reflects true medication use, cumulative exposure, and patient risk. Prior literature supports the use of intranasal and systemic corticosteroids in chronic rhinosinusitis with nasal polyps, but systemic corticosteroids have well-recognized adverse effects when repeated or cumulative exposure is high [5-7]. The present analysis measured whether a prescription occurred, not the number of prescriptions, dose, duration, adherence, or total corticosteroid exposure. The data therefore support a difference in observed post-FESS prescribing frequency, but not a conclusion about cumulative corticosteroid burden.

Prior literature also shows why the present outcome definitions should remain modest. Nasal polyp recurrence after FESS may require long-term follow-up and objective assessment [13]. Postoperative systemic corticosteroid trials, postoperative care reviews, surgical inflammation literature, steroid-impregnated spacer studies, and steroid-eluting implant analyses all support the broader concept that postoperative inflammatory control is clinically important, but they do not establish that surgical sequencing alone reduces inflammation or steroid burden [14-18]. Studies of concurrent sinonasal and nasal structural surgery also support the feasibility of combined procedures in selected patients, reinforcing that same-day surgery should not be characterized as inherently harmful [19,20].

This study has several strengths. It used a large real-world multicenter dataset, a clearly defined surgical population, and propensity score matching to reduce measured imbalance between same-day and staged cohorts. It also evaluated both medication prescribing and a longer-term coded nasal polyp diagnosis outcome, allowing postoperative management patterns to be considered alongside a structured disease-related endpoint.

Several limitations should be noted. This study was retrospective and observational, so residual confounding and confounding by indication cannot be excluded despite propensity score matching. Patients selected for same-day versus staged surgery may have differed in disease severity, anatomy, comorbidity burden, surgeon preference, patient preference, insurance or reimbursement factors, access to care, and postoperative management patterns. The staged cohort was defined by a minimum separation of 30 days, but the analysis did not specify an upper limit between septoplasty and FESS. Long intervals may represent clinically distinct procedures rather than a planned staged strategy, making the findings sensitive to the broad operational definition of staged surgery. TriNetX data depend on structured diagnosis, procedure, and medication codes, which may be incomplete or inconsistently documented across healthcare organizations. Although exclusion-code descriptions were reviewed for coding accuracy, code-based exclusions may not fully capture all relevant clinical conditions. Medication data reflect prescribing or ordering rather than confirmed dispensing, adherence, dose, duration, or cumulative corticosteroid exposure. Steroid use between septoplasty and FESS in the staged cohort was not captured in the post-FESS outcome window, so total steroid exposure across the full staged-care pathway may be underestimated. The database also does not provide symptom severity, endoscopic findings, radiographic scores, operative details, surgeon decision-making, insurance constraints, reasons for staging, or postoperative treatment protocols. Finally, the staged cohort was much smaller before matching, which may limit generalizability.

Overall, these findings suggest that same-day septoplasty and FESS are associated with higher postoperative corticosteroid prescribing, while coded postoperative nasal polyp diagnosis does not differ significantly from staged surgery. The results should be interpreted as hypothesis-generating and should not be used to conclude that staging reduces inflammatory burden or that same-day surgery worsens disease control. Future studies should evaluate clinically bounded staging intervals, include surgery dates and interval distributions, quantify total corticosteroid dose and number of prescriptions, capture medications used between staged procedures, and incorporate endoscopic, radiographic, and symptom-based measures of disease severity.

## Conclusions

In this retrospective, observational, propensity score-matched TriNetX cohort, same-day septoplasty and FESS were associated with a higher rate of recorded postoperative systemic and intranasal corticosteroid orders (prescribing) than staged septoplasty and FESS. Because the outcome was based on recorded medication orders rather than confirmed dispensing, adherence, dose, or duration, this difference should be interpreted as a difference in observed prescribing frequency rather than in true corticosteroid use or exposure. Coded postoperative nasal polyp diagnosis did not differ significantly between groups over two years.

Given the retrospective design and prescription-based outcomes, these findings are hypothesis-generating and should not be interpreted as causal evidence that staged surgery reduces total corticosteroid burden or that either surgical strategy provides equivalent endoscopic disease control. The lack of an upper limit for the staged interval is an important limitation, and the results may be sensitive to how staged surgery is defined. Surgical timing decisions should remain individualized and should account for disease severity, anatomy, patient preference, operative efficiency, reimbursement context, and the clinical importance of minimizing corticosteroid exposure.
